# *In Silico* Exploration of 1,7-Diazacarbazole Analogs as Checkpoint Kinase 1 Inhibitors by Using 3D QSAR, Molecular Docking Study, and Molecular Dynamics Simulations

**DOI:** 10.3390/molecules21050591

**Published:** 2016-05-05

**Authors:** Xiaodong Gao, Liping Han, Yujie Ren

**Affiliations:** School of Chemistry and Environmental Engineering, Shanghai Institute of Technology, Shanghai 201418, China; m15201835052_2@163.com (X.G.); hanliping108@163.com (L.H.)

**Keywords:** Chk1 protein, docking, CoMFA, CoMSIA, molecular dynamics

## Abstract

Checkpoint kinase 1 (Chk1) is an important serine/threonine kinase with a self-protection function. The combination of Chk1 inhibitors and anti-cancer drugs can enhance the selectivity of tumor therapy. In this work, a set of 1,7-diazacarbazole analogs were identified as potent Chk1 inhibitors through a series of computer-aided drug design processes, including three-dimensional quantitative structure–activity relationship (3D-QSAR) modeling, molecular docking, and molecular dynamics simulations. The optimal QSAR models showed significant cross-validated correlation q^2^ values (0.531, 0.726), fitted correlation r^2^ coefficients (higher than 0.90), and standard error of prediction (less than 0.250). These results suggested that the developed models possess good predictive ability. Moreover, molecular docking and molecular dynamics simulations were applied to highlight the important interactions between the ligand and the Chk1 receptor protein. This study shows that hydrogen bonding and electrostatic forces are key interactions that confer bioactivity.

## 1. Introduction

Cancer refers to uncontrolled abnormal cell division with propensity for tissue invasion and is a highly unique genomic disease [1]. Everyone is born with a unique set of genomes, which determine the risk of cancer. After its onset, the genetic disease quickly causes other genomic mutations that lead to cancer. Treatments for cancer are mainly categorized as chemotherapy, radiotherapy, and surgery. DNA-damaging agents are introduced in chemical treatments for cancer to disturb the chromosome structural integrity or disrupt DNA metabolism, synthesis, and transcription. This method affects both tumor and normal cells; hence, anti-cancer drugs show the lowest therapeutic index among other anti-cancer therapies. However, DNA-damaging agents exert a mutual inhibiting effect on normal cells. The effectiveness of treatment for DNA damage may be improved by inhibiting cell-cycle checkpoint kinases, which can facilitate cell-cycle arrest and provide time for lesion repair.

**Figure 1:**
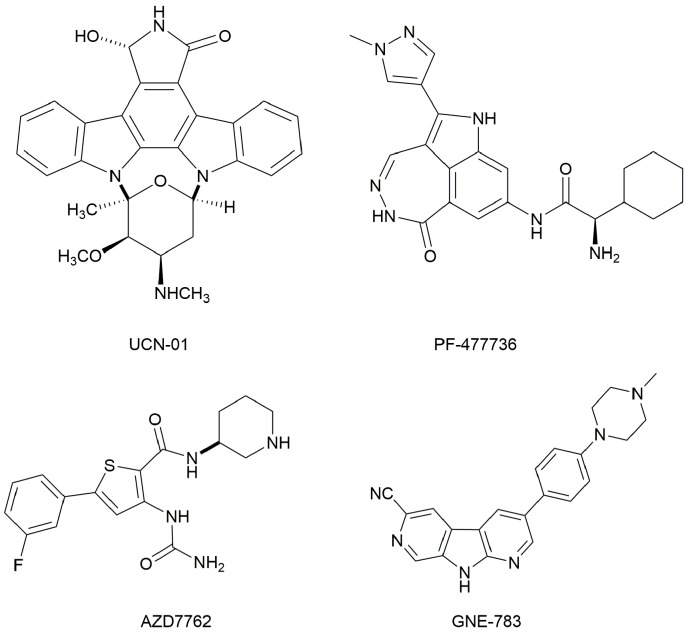
The protein Chk1 inhibitors.

Checkpoint kinase 1 (Chk1) is an important serine/threonine kinase with a self-protection function [2]. DNA damage can activate the Chk1 protein through the ATR/Chk1 signal transduction pathway. Consequently, the activated Chk1 causes cell-cycle arrest and repairs gene transcription, which ensures the integrity and stability of the genome [3,4]. Cancer cells are accompanied by genetic deficiencies in the P53 gene, leading to the lack of the G1 cell-cycle checkpoint [5]. Therefore, most cancer cells repair themselves through the S and G2–M checkpoint. Chk1 inhibition can disable the function of the S and G2 checkpoint, thereby impeding cancer cell repair and resulting in mitosis disorders, and even cell death or apoptosis. Normal cells possess a relatively complete cycle checkpoint function and are relatively insensitive to Chk1 inhibitors [6]. Currently, clinical studies evaluated several Chk1 inhibitors (Figure 1), such as UCN-01 [7], PF-477736 [8], and AZD7762 [9,10]. These inhibitors, in combination with other anti-cancer drugs, can improve the effect of cancer treatment. For example, AZD7762 can enhance the selectivity of gemcitabine for P53-deficient cells. Thus, the development of Chk1 inhibitors has been a highly active area of research in cancer treatment. Recent works reported a series of potential candidates, especially GNE-783 [11,12,13]. This inhibitor shows a significant half maximal inhibitory concentration (IC_50_) value of 1.3 nM against the Chk1 protein. Furthermore, GNE-783 demonstrates good oral availability. However, *in vivo* studies revealed that GNE-783 potently binds to acetylcholine esterase (AChE) and produces muscle fasciculation, thereby interrupting the development of this compound as a therapeutic agent. Gazzard *et al.* [14] synthesized a novel series of GNE-783 analogs with oral availability to obtain superior bioactive compounds. The influence regularity of AChE bioactivity in AChE binding mode was described. This report discussed that low binding forces in the complex between the AChE protein and its analogs achieve low AChE inhibitor activity. Meanwhile, biological evaluation obtained satisfactory results in the structure modification of GNE-783 analogs. GNE-145 (compound **17**, Table 1) shows significant IC_50_ values of 2.5 nM and 2.42 μM against the Chk1 protein and AChE, respectively. These results indicate that this series of compounds include potent Chk1 inhibitors with low AChE bioactivity.

*In silico* modeling technology is widely used in drug discovery [15,16,17,18] and chemical field. The design of novel drugs [19] is difficult to achieve without computational chemistry tools because experimentation procedures are expensive and complicated. These computational tools include molecular docking [20], 3D-QSAR, and molecular dynamics simulations, which can be used to understand the relationship between chemical structure and inhibitory activity and develop novel drug candidates. For example, Veselinovića *et al.* [21] used Monte Carlo QSAR models for predicting the organophosphate inhibition of AChE. Caballero *et al.* [22] used docking and QSAR models to study the quantitative structure–activity relationships of imidazo[1,2-*a*]pyrazines derivatives as Chk1 inhibitors. These studies demonstrated the potential feasibility of theoretical calculations. In this work, linear regression analysis methods, such as comparative molecular field analysis (CoMFA) and comparative molecular similarity index analysis CoMSIA), while considering molecular interaction fields were used for *in silico* identification of 1,7-diazacarbazole analogs as Chk1 inhibitors. The developed models enable detailed examination of molecular structural factors that affect bioactivity. Moreover, these models can predict the bioactivities of new analogs. Molecular docking and dynamics simulations illustrate the possible binding modes of a certain structure and its receptor protein. These binding modes describe that hydrogen bonding and electrostatic forces significantly contribute to bioactivity.

## 2. Materials and Methods

### 2.1. Dataset

The dataset used for molecular modeling studies contains 40 compounds which were designed and biological evaluation by Gazzard [14] to explore new 1, 7-diazacarbazole analogs as potent Chk1 inhibitors. The structures of the analogues as well as the pIC_50_ values (pIC_50_ = −logIC_50_) are described in Table 1. The experimental data obtained are randomly divided into a training set (35 structures) for QSAR model generation, and the remaining five molecules constituted the test set for model validation. A previous study [23] enumerated feasible and effective verification methods, and the random test set is an important component for ensuring the accuracy of the method.

### 2.2. Energy Minimization and Modeling Alignment

All the structures were constructed using the 2D sketcher module in Sybyl-X 2.0 molecular modeling package. Minimum energy calculation of all structures was performed using the Tripos force field [24], followed by 10,000 iterations. The atomic point charges were calculated using the Gasteiger-Hückel [25] method. The root mean square (RMS) of the gradient was set to 0.005 kcal/(mol·Å) [26].

**Figure 2:**
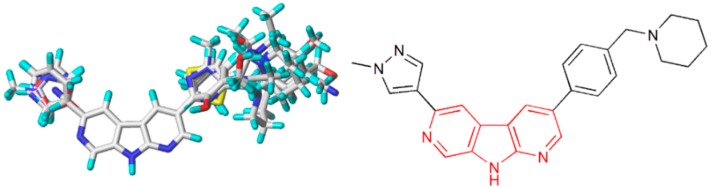
Common substructure (red) used in alignment, and the alignment of training structures.

The minimum energy conformation selection and the alignment rule are two crucial factors to build an ideal model. In general, two alignment methods were used to derive the reliable model, including the maximum common substructure (MCS) alignment and the docking-based alignment. In this study, the MCS alignment rule was used to complete the molecular alignment. CoMFA and CoMSIA approaches aligned the structures to compound **28**, which is assumed to be the highest bioactive conformation. The common structure (red) was used to position the rest of the compounds and the alignment of the training structures were shown in Figure 2.

### 2.3. Generation of the QSAR Model

In this study, CoMFA and CoMSIA methods were used to construct 3D-QSAR models. Both CoMFA and CoMSIA methods were based on the field concepts which were around the aligned molecules. The CoMFA model calculated the steric and electrostatic fields [27], and the CoMSIA method calculated five different similarity fields, including steric (S), electrostatic (E), hydrophobic (H), H-bond donor (D), and H-bond acceptor (A) fields [28]. The pIC_50_ values were used as dependent variables to characterize the molecular structure, and the other parameters were set by default.

### 2.4. Partial Least Squares (PLS) Analysis and Validation of the QSAR Models

The quantitative relationships between the molecular descriptors and bioactivities were established by partial least squares (PLS) regression analysis [29]. There are many methods used for cross-validation analysis, including leave-one-out (LOO), leave-many-out (LMO), or leave-N-out (LNO) cross-validation, y-randomized validation, and bootstrapping method. The LOO cross-validation method was used in this study to measure the quality of the model. Then, the cross-validation correlation coefficient (q^2^) value, the correlation coefficient (r^2^) value, the optimum number of components (ONC) values, the standard error of estimate (SEE), and the F-statistic values were obtained. These statistics were the results of cross-validation, which given enough information about the predictive abilities of the model. The q^2^ values were usually used to measure the impression of how predictive the model is. The test set was used to evaluate the capacity of external validation. Different statistics and methods can evaluate the predictive power of the model. For example, the external predictive correlation coefficients (r^2^_pred_ values) [30,31,32] and four criteria proposed by Golbraikh [33]. Then, the statistics’ r^2^_pred_ values were applied in this study, and the r^2^_pred_ values are calculated using the following Equation (1):

(1) $$r_{\text{pred}}^{2}=1-\frac{\text{PRESS}}{\text{SD}}$$

The SD value is the sum of squared deviation between pIC_50_ values of test set compounds and the mean pIC_50_ of the training set structure. The PRESS value is the sum of squared deviations between the actual and the predicted bioactivities of the test compounds.

### 2.5. Molecular Docking Simulations

Surflex-Dock module implemented in Sybyl-X 2.0 was used for the molecular docking studies in this work. The crystal structures of ChK1 kinase domain were downloaded from the Protein Data Bank (PDB ID: 4RVK) [14]. The hydrogen atoms were added, as well as the water molecules, and the ligands had been deleted. The other parameters were default in the software. Subsequently, each structure was docked into the activity pockets for further analysis. An item from the docking results obtained at least 20 ratings, and the highest scoring conformation was studied.

## 3. Results and Discussions

### 3.1. Statistical Analysis and Validation

CoMFA and CoMSIA approaches were carried out using the 40 1,7-diazacarbazole derivatives chosen in the Chk1 inhibition experiment. The statistical summaries are shown in Table 2. In the CoMFA model, the PLS regression analysis yielded a q^2^ of 0.726 with SEE of 0.215. Then, the non-cross-validated method gave an r^2^ value of 0.918, an F-values of 115.292 and three optimal components.

In the CoMSIA model, a q^2^ value of 0.531 and r^2^ value of 0.950 were obtained when five field descriptors were considered. The F-value, SEE value, and ONC values were 141.412, 0.171, and 4, respectively. From the field contribution results, we found that the electrostatic field played a major contribution among the present fields. These data also showed the reliability of the CoMSIA model.

**Figure 3:**
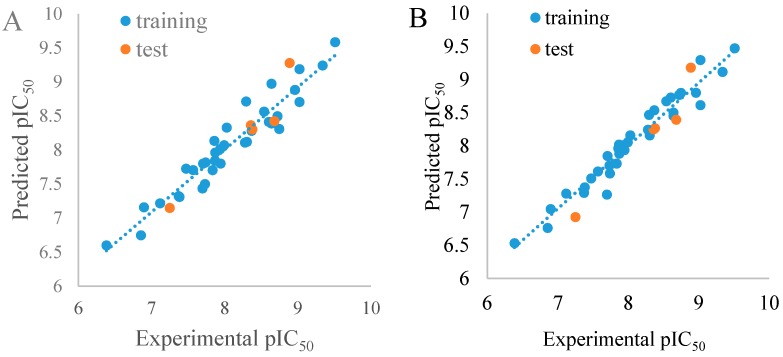
Plots of experimental *vs.* predicted pIC_50_ values for the total set in the CoMFA (**A**) and CoMSIA (**B**) models.

A test set of 40 compounds were used to validate the accuracy and predictability of the model. The values of r^2^_pred_, shown in the Table 2, were 0.878 for CoMFA and 0.846 for the CoMSIA model. These statistical indices indicated a good external predictive capacity of the models. The graphs showing the experimental and predicted pIC_50_ values for the total set used in the CoMFA and CoMSIA approaches are described in Figure 3. The good linear relationships illustrated that the bioactivities predicted by the derived models were in agreement with the experimental data, indicating that these models had satisfactory predictive capacity.

### 3.2. CoMFA/CoMSIA Contour Map Analysis

The steady 3D QSAR models were generally applied to drug discovery processes to predict the biological activities of unknown derivatives. Moreover, the effects of the field descriptors contributing to activities can be partitioned and visualized through 3D contour plots. The field type of the contour plot was set to StDev × Coeff to aid visualization.

**Figure 4:**
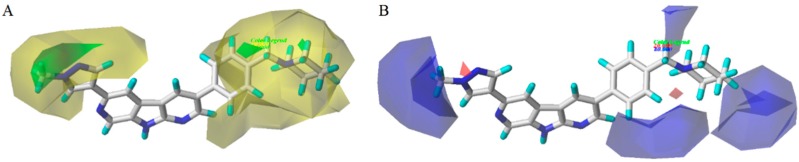
Steric (**A**) and electrostatic (**B**) contours of the CoMFA model. The green color shows the favored steric area and the yellow color show steric area. The red color shows the favored negative electrostatic area and the blue color shows the favored positive electrostatic area.

The CoMFA contour maps are illustrated in Figure 4. These contours plots demonstrate regions where the steric and electrostatic variations in the different molecular features lead to either increased or reduced bioactivity. The most structure **28** was chosen as a reference to aid visualization. Two green regions and two yellow places exist around the compound zones represented the steric favorable and unfavorable areas, respectively. The green maps around the methyl group of pyrazole ring indicated that bulk groups were favored there. The methyl group of pyrazole ring at this position may be favorable to the interaction between the compound and its receptor. It can be explained by the fact that the bioactivity of compound **28** (pIC_50_ = 9.51) was better than those of compound **27** (pIC_50_ = 8.56). A yellow contour around the R_1_ substituent indicated that the small bulky group can enhance bioactivities. This phenomenon could be concluded by comparing the activities between compounds **25** (pIC_50_ = 9.34) and **30** (pIC_50_ = 8.64).

In the CoMFA electrostatic contour maps (Figure 4), the regions in red implied where electronegative groups improved activities, whereas the positions in blue purported where electropositive groups enhanced activities. A blue contour around the methyl group of pyrazole ring, indicating a positive atomic charge group in this position had a positive effect on the inhibitory activity, such as compounds **28** and **27**. Moreover, a large blue tetrahedron around the para-position of the piperidine ring revealed the importance of positive atomic groups. For example, compounds **11**, **13**, **14**, and **15** shown moderate activity probably because the para-position of piperidine ring was replaced by electronegative groups. Simultaneously, two small red cubes near the nitrogen atoms of the pyrazole and piperidine rings indicated that the negatively-charged groups were helpful for inhibitory activity, which was consistent with the experimental results.

**Figure 5:**
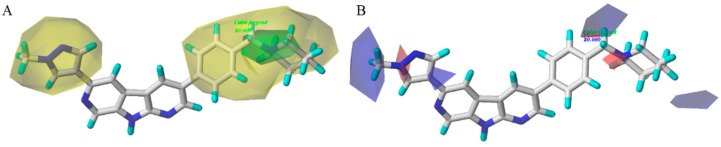
Steric (**A**) and electrostatic (**B**) contours of the CoMSIA model.

**Figure 6:**
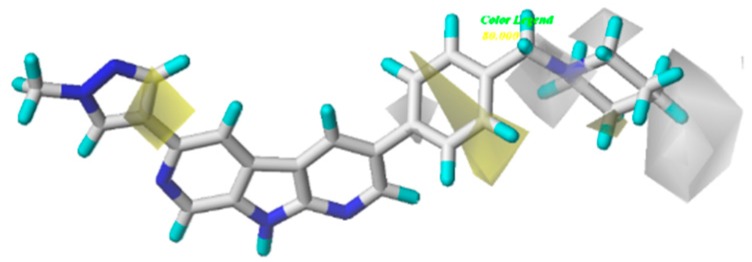
Hydrophobic contours of the CoMSIA model. The yellow color shows the favored hydrophobic area, the white color shows the disfavored hydrophobic area.

The CoMSIA steric and electrostatic contour plots were described in Figure 5. Not surprisingly, most contours were similar to those of CoMFA model and, hence, were not discussed. The hydrophobic contour plot was constructed through CoMSIA model, presented in Figure 6. The yellow and gray colors represented favorable and unfavorable hydrophobic areas, respectively. The hydrophobic contour maps were exclusively located around R_1_ and R_2_ substituents. A small yellow cube surrounding the C-1 position of pyrazole ring (R_1_ group) indicated hydrophobic groups were advantageous for inhibitory activity. Compounds **25**–**29** showed better inhibitory activities than compounds **9**–**15**, probably because –CN group was replaced by hydrophobic substituents. A yellow cone around the meta-position of benzene ring revealed that hydrophobic substituents in this region produced increased bioactivity. The gray contours around the para-position of the piperidine ring (R_2_ substituent) suggested that the hydrophilic group in this region could increase activity. It can be demonstrated by the fact that the biological activity of compound **11** (pIC_50_ = 8.745) was slightly higher than that of compound **13** (pIC_50_ = 8.301).

**Figure 7:**
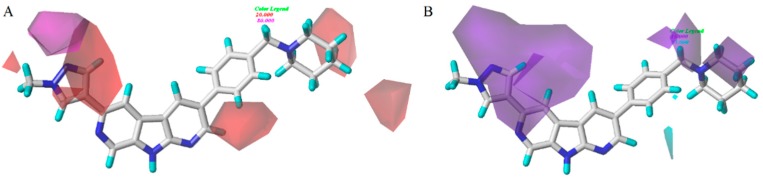
Hydrogen bonding contour (include H-bond acceptor (**A**) and H-bond donor (**B**) contour plots) of CoMSIA model. The magenta color shows the favored H-acceptor area, the red color shows the disfavored H-acceptor area, the cyan color shows the favored H-donor area, and the purple color represents the disfavored H-donor area.

The results of the statistical analysis revealed that H-bond acceptor field had a significant contribution on the contour maps. The H-bond acceptor contour plots of the CoMSIA model containing the most active compound **28** were illustrated in Figure 7A. The magenta contour around the N atom of the pyrazole ring indicated that the hydrogen bond acceptor group was conducive to the improvement of bioactivity. The nitrogen atom in this position may be beneficial to the generation of the hydrogen bond between the compound and its receptor protein. It can also be proved by the fact that compounds **25**–**28**, which displayed higher activities than other compounds. Thus, the H-bond acceptor group was believed to have a strong bioactivity on Chk1. As shown in Figure 7A, the regions in red implied where hydrogen bond donor group decrease activity. A large red tetrahedron surrounding the para-position of piperidine ring indicated that the hydrogen bond donor group was adverse to inhibitory activity, which can be demonstrated by the fact that compounds **11** and **13** showed a satisfactory activity. The other red contours were away from the most potent compound **28** and, hence, are not discussed. A prominent purple contour around the piperidine ring (R_1_ substitute) indicated that an H-bond donor was adverse to bioactivity (Figure 7B). For example, the order of many compounds’ bioactivities was: **28** > **24** > **21**. Compounds **21** and **24** with hydrogen bond donor groups showed low activities, which was in agreement with the contour map. The cyan color around the N-1 position of piperidine ring (R_2_ substitute) indicated that the hydrogen bond donor was favorable to activity. This result was the same to that of the hydrophobic contour plot.

According to the conclusions above, six new compounds (**2a**–**2g**) with satisfactorily predicted pIC_50_ values have been designed shown in Table 3. These designed compounds exhibited satisfactory predictive values also indicated the correctness of CoMFA and CoMSIA models.

### 3.3. Molecular Docking

**Figure 8:**
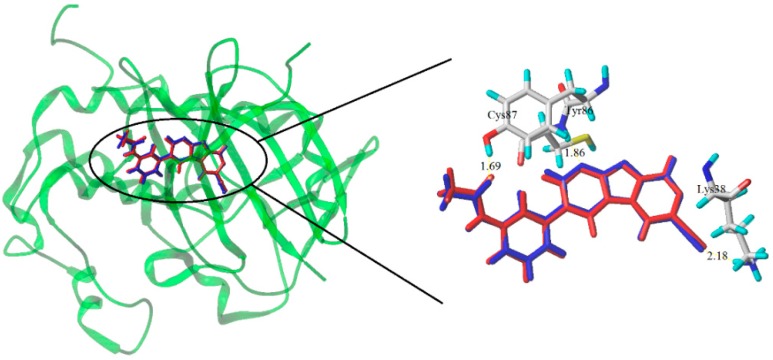
Re-docking results of the compound into the binding site of Chk1 protein and the crystal structure (**blue ribbon**) for the compound complex. The re-docked ligand (**blue**) and the original ligand (**red**) shown as a stick model. The hydrogen-bonds are shown as yellow lines, with distance unit of Å.

Molecular docking protocols were widely used to investigate the possible binding modes between the target derivatives and the receptor protein, which aided in the understanding the QSAR revealed by CoMFA/CoMSIA models. Prior to docking, a re-docking simulation was applied to validate the accuracy of molecular docking. The target ligand taken from the crystal structure was re-docked into the active site with the root mean square deviation (RMSD) value of 0.225 Å for 4RVK. As shown in the Figure 8, the region of re-docking ligand (blue) was same to that of the original ligand (red). Three hydrogen bonds which were formed between the ligand and the protein appeared on the horizon. The H-bond distances were observed to be 1.69 Å (C=O···H-Cys87), 1.86 Å(C=N···H-Tyr86), 2.18 Å (CN···H-Lys38), respectively. In short, the surflex-dock program could successfully reproduce the original conformation.

**Figure 9:**
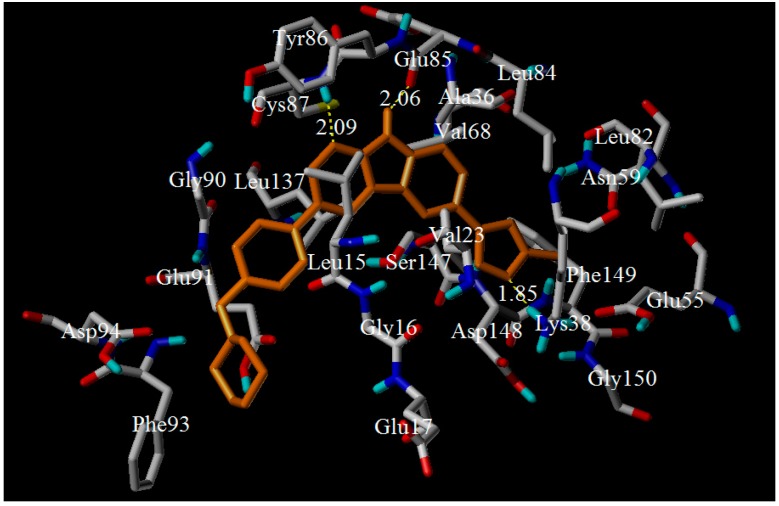
Docking result of compound **28** in the activity site of Chk1 protein. Ligand (yellow) and the key residues colored white for binding interaction were shown as stick model. The hydrogen-bonds were described as yellow lines, with distance unit of Å.

Subsequently, the most compound **28** was docked into the ligand-binding pocket of Chk1 protein. As described in Figure 9, the docking results demonstrated three hydrogen bonds between compound **28** and the key residues (including Lys38, Glu85 and Cys87) in the Chk1 active pocket. The nitrogen atom of 1,7-diazacarbazole provided a hydrogen-bonding with Tyr86 (2.09 Å), the N–H group was hydrogen bonded to residues Glu85 (2.06 Å), and the 4-N of pyrazole ring formed a hydrogen-bonding with Lys38 (1.85 Å). These residues could also interact with the target compound through electrostatic interactions. Moreover, the other residues, such as Tyr86, Glu91, Phe149, Asp148, and Glu55, could also stabilize ligand through electrostatic interaction. The CoMFA/CoMSIA electrostatic-favorable blue contour around the pyrazole and the hetero atom further supported the structure-based analysis. In addition, Van der Waals forces were formed between the compound and important residues (for example: Ser147, Val68, Leu84, Asn59, Gly150, Leu82, Gly90, Ala36, Leu15, Leu137, Val23, Asp94, Phe93, Gly16, and Glu17). These van der Walls interaction was involved in the activities of stabilizing compound in the active pocket. The docked model revealed that the hydrogen-bonding/electrostatic interactions played an important role in the interaction between the inhibitor and the protein, and the hydrogen bonds were similar to those in literature [11]. Moreover, the results obtained by the docking had been compared with the QSAR results to verify mutually. These interactions match well with the results of H-bond acceptor/electrostatic contour maps.

**Figure 10:**
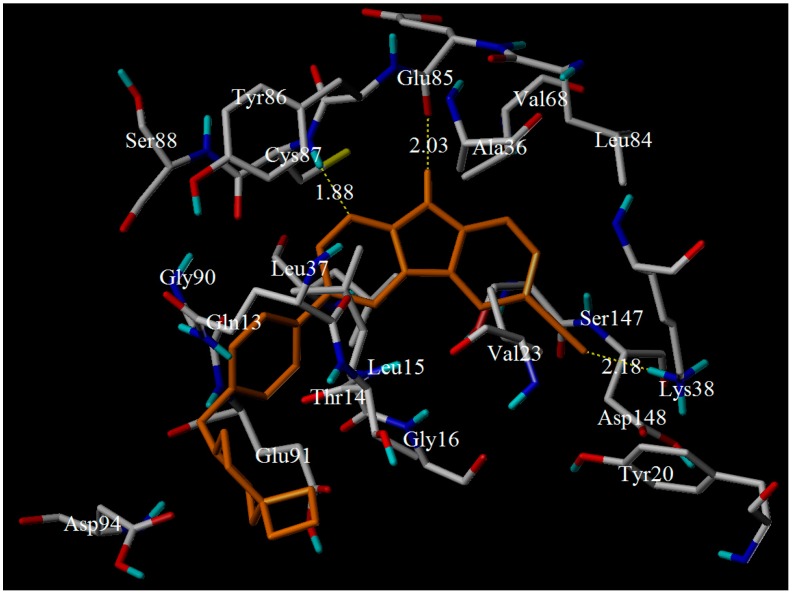
Docking result of compound **17** in the activity site of Chk1 protein. Ligands (yellow) and the key residues (white) for binding interactions were shown as a stick model. The hydrogen bonds were described as yellow lines, with distance measured in Å.

Compound **17** as a potential candidate was docked into the same active site to further analyze the impact of residues on the inhibitor activity. From the Figure 10, three hydrogen bonds were in sight. The H-bond distances were observed to be 1.88 Å (C–N∙∙∙H–Cys87), 2.03 Å (N-H···O-Glu85), and 2.18 Å (CN···H-N-Lys38), respectively. These H-bonds were the same to that of compound **28**. Furthermore, electrostatic interaction was formed between this compound and the important residues (e.g., Tyr20, Asp148, Lys38, Glu85, Tyr86, Cys87, Thr14, and Leu15). These residues were mainly around R_1_ substituent. It could also confirm the importance of the electrostatic fields to bioactivity. Of course, the van der Waals force was essential. The amino residues, such as Ser147, Leu37, Val23, Glu91, Gly16, Asp94, Leu84, Val68, Ala36, Gly90, Ser88 and Gln13, could form van der Waals interaction with the target compound. These results also suggested that hydrogen bonding and electrostatic forces were the key interaction that confer bioactivity.

### 3.4. Molecular Dynamics Simulations

In a subsequent step, the molecular dynamics process was performed on compounds **28** and **17** to further explore the probable binding modes between the compounds and the receptor protein. Complex Chk1 structure of the compound as generated by the previous docking modelling was used as the initial coordinates for molecular dynamics study. The molecular dynamics simulations were performed using the dynamics module of SYBYL-X 2.0 at the vacuum environment [34,35]. Then, the energy minimization was performed for the complex molecule with Gasteiger-Hückel charge and Tripos force field without water using Boltzmann initial velocity. The simulations were executed using normal temperature and volume (NTV) [36] ensemble 300 K with coupling 100 fs. Additionally, we perform a 5 ns simulation with a time step of 1 fs and snapshot the conformation every 1000 fs.

**Figure 11:**
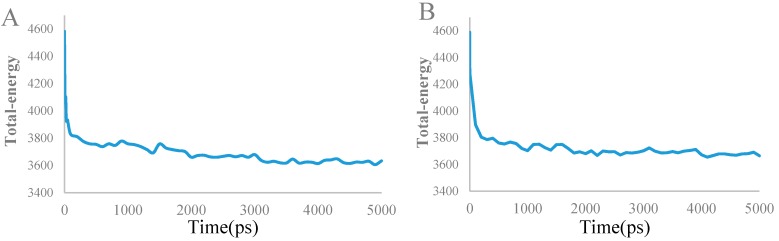
Plot of the total-energy docked complex (compound **28** (**A**) and compound **17** (**B**)) and the molecular dynamics simulation time (ps) in the molecular dynamics simulated structure.

**Figure 12:**
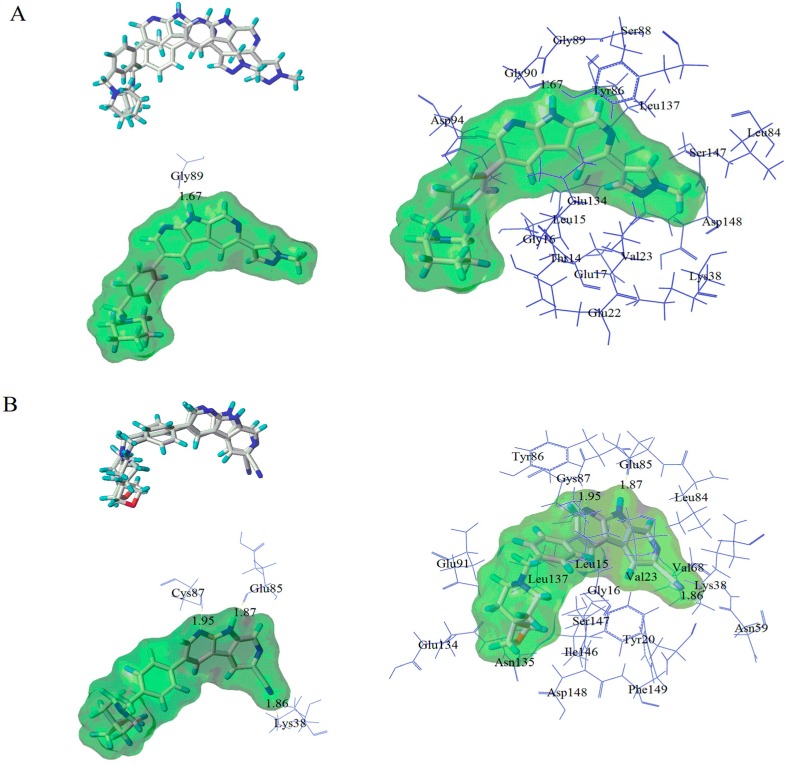
The alignment of molecular dynamics simulated and original ligand, as well as the docking results of compound **28** (**A**) and **17** (**B**).

A 5 ns simulation of the complex between the compound **28** and the protein was run to energy balance at 2 ns to obtain the stable conformation. The total energy of compound ranging from 4057 to 3620 KJ/mol was illustrated in Figure 11A. After 2 ns, the total energy of the complex dropped to 3620 KJ/mol, and tended to stability. This result suggested that the ligand-protein complex could reach the metastable conformation after 2 ns of simulation. The alignment of original and molecular dynamics simulated ligand were shown in Figure 12A to indicate a high similarity among these two ligand. From the Figure 12A, some residues (such as: Tyr86, Ser147, Gly90, Asp94, Asp148, Glu17, Leu15, Leu137, Leu84, and Gly16) were still important to the interaction between compound **28** and Chk1 protein. However, the number of hydrogen bonds and the amino acid residues decreased. One hydrogen bond was formed between Gly89 and compound **28**, with the distance of 1.67 Å. This reside formed not only hydrogen-bonding to ligand, but also formed the electrostatic force. Moreover, most residues (such as: Asp148, Glu17, Leu15, *et al.*) were bound to the target compound through the electrostatic interaction. Therefore, we predict that the electrostatic interaction has a greater impact on activity of compound **28**. These results match well with the field contribution of CoMSIA model.

The docked complex of compound **17** and the Chk1 protein was studied subsequently through a 5 ns simulation. The total energy of the docked complex was shown in Figure 11B. From Figure 11B, the total energy tends to be stable after 1 ns. The alignment of molecular dynamics simulated and the original ligand was described in Figure 12B. Meanwhile, the key residues which interacted with the target compound were in sight. Some key residues (Glu85, Cys87, and Lys38) still interacted with compounds through H-bonding and electrostatic force. The H-bond distances were 1.87 Å, 1.95 Å, and 1.86 Å, respectively. Combining the docking results, the key residues (Glu85, Cys87, and Lys38) were predicted to be the important factor influencing the bioactivity of Chk1.

## 4. Conclusions

In this work, several 1,7-diazacarbazole analogs were identified as potentially effective oral Chk1 inhibitors through a series of computer-aided drug design processes, such as 3D-QSAR modeling, molecular docking, and molecular dynamics simulations. The CoMFA/CoMSIA models with statistical capacity showed good internal and external validation abilities and can be used to predict new and potential molecules. Moreover, the obtained contour maps can be used to guide the design of new compounds with high Chk1 inhibitory activity. Meanwhile, molecular docking and molecular dynamics process were established to study the possible binding modes of inhibitors at the active pocket of Chk1. Some key residues, such as Glu85, Cys87, Lys38, and Gly90, were found. Hydrogen bonding and electrostatic forces were predicted to be the key interactions that confer bioactivity. Overall, these results show that the optimal CoMFA/CoMSIA models can be used to predict novel Chk1 inhibitors and guide the development of new potential oral analogs.

## Figures and Tables

**Table 1:** Chemical structural formulas of all structures. Statistical parameters of the actual and predicted bioactivity by CoMFA and CoMSIA, as well as the residual between the actual and predicted pIC_50_ values. All the aligned molecular dataset used for the 3D QSAR studies were shown in Table S1 in the supplementary materials. 
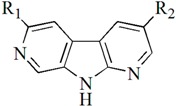

NO.	R_1_	R_2_	Actual pIC_50_	CoMFA		CoMSIA	
				pIC_50_	Residual	pIC_50_	Residual
1	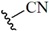	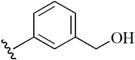	7.567	7.706	−0.139	7.614	−0.047
2	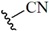	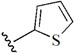	7.695	7.437	0.258	7.266	0.429
3	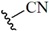	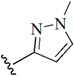	7.471	7.726	−0.255	7.509	−0.038
4	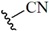	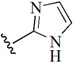	7.833	7.705	0.128	7.732	0.101
5	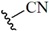	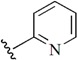	6.896	7.160	−0.264	7.046	-0.150
6	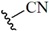	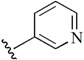	7.116	7.219	-0.103	7.280	−0.164
7	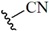	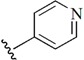	7.370	7.323	0.047	7.292	0.078
8	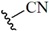	H	6.383	6.600	−0.217	6.532	−0.149
9	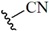	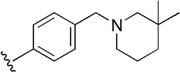	8.027	8.330	−0.303	8.158	−0.131
10	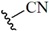	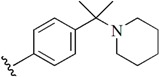	7.991	8.072	−0.081	8.055	−0.064
11	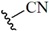	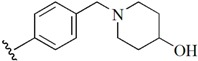	8.745	8.310	0.435	8.799	−0.054
12	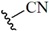	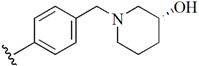	8.721	8.497	0.224	8.769	−0.048
13	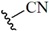	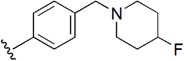	8.301	8.121	0.180	8.159	0.142
14	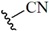	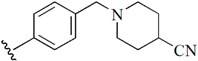	7.936	8.014	−0.078	8.012	−0.076
15	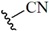	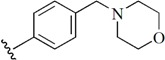	7.857	8.136	−0.279	7.962	−0.105
16	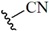	Br	6.854	6.748	0.106	6.762	0.092
17	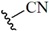	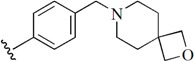	8.602	8.414	0.188	8.729	−0.127
18	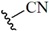	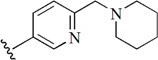	8.276	8.110	0.166	8.244	0.032
19	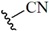	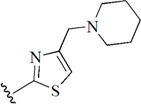	7.866	7.843	0.023	8.019	−0.153
20	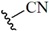	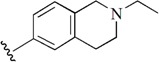	8.638	8.390	0.248	8.502	0.136
21	H	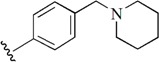	7.730	7.500	0.230	7.703	0.027
22	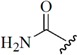	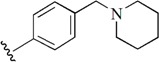	8.367	8.283	0.084	8.537	−0.170
23	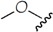	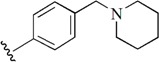	7.703	7.802	−0.099	7.847	−0.144
24	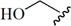	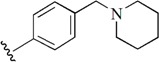	7.738	7.818	−0.080	7.583	0.155
25	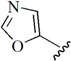	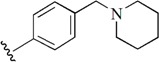	9.337	9.241	0.096	9.116	0.221
26	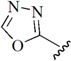	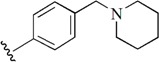	9.022	9.189	−0.167	9.295	−0.273
27	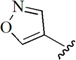	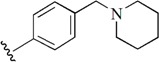	8.959	8.883	0.076	8.800	0.159
28	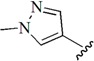	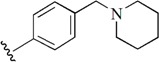	9.509	9.585	−0.076	9.471	0.038
29	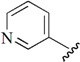	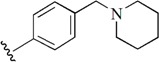	9.022	8.706	0.316	8.615	0.407
30	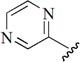	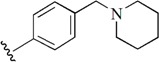	8.638	8.974	−0.336	8.452	0.186
31	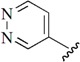	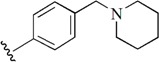	8.538	8.565	−0.027	8.672	−0.134
32	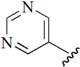	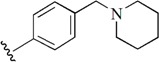	8.292	8.713	−0.421	8.468	−0.176
33	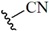	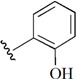	7.380	7.311	0.069	7.374	0.006
34	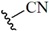	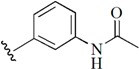	7.870	7.963	−0.093	7.882	−0.012
35	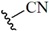	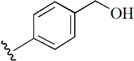	7.943	7.802	0.141	7.937	0.006
Test1	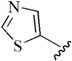	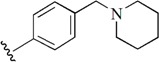	8.886	9.278	−0.392	9.180	−0.294
Test2	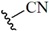	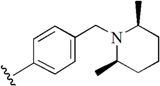	8.678	8.425	0.253	8.393	0.285
Test3	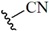	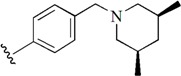	8.377	8.304	0.073	8.266	0.111
Test4	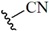	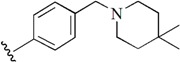	8.357	8.364	−0.007	8.251	0.106
Test5	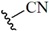	NHEt	7.247	7.151	0.096	6.927	0.320

**Table 2:** The PLS statistical results of CoMFA and CoMSIA model.

PLS Statistics	CoMFA	CoMSIA
q^2 a^	0.726	0.531
NOC ^b^	3	4
r^2 c^	0.918	0.950
SEE ^d^	0.215	0.171
F ^e^	115.292	141.412
r^2^_pred_^f^	0.878	0.846
Steric	0.509	0.199
Electrostatic	0.491	0.283
H-acceptor	-	0.238
H-donor	-	0.099
Hydrophobic	-	0.182

^a^ Leave-one-out cross-validated correlation coefficient; ^b^ the optimum number of components; ^c^ Non-cross-validated correlation coefficient; ^d^ Standard error estimate of non-cross-validated correlation coefficient; ^e^ F-test value; ^f^ the external predictive correlation coefficients.

**Table 3:** Designed molecules and predicted inhibit activities values of Chk 1 through CoMFA and CoMSIA. 
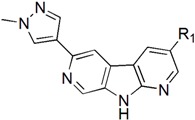

No.	R_1_	Predicted pIC_50_ by CoMFA	Predicted pIC_50_ by CoMSIA
**2a**	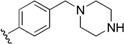	9.570	9.682
**2b**	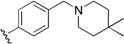	9.654	9.504
**2c**	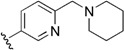	9.412	9.483
**2d**	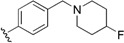	9.411	9.400
**2f**	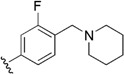	9.619	9.297
**2g**	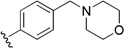	9.424	9.201

## Supplementary Materials

Supplementary materials can be accessed at: http://www.mdpi.com/1420-3049/21/5/591/s1.
