# Supplementary material for: In Silico Exploration of 1,7-Diazacarbazole Analogs as Checkpoint Kinase 1 Inhibitors by Using 3D QSAR, Molecular Docking Study, and Molecular Dynamics Simulations
Source: Molecules. 2016 May 5;21(5):591. doi: 10.3390/molecules21050591 (PMC6273173; doi:10.3390/molecules21050591)
Supplement: Supplementary file 1 [file molecules-21-00591-s001.pdf]

Supplementary Material: *In Silico* Exploration of 1,7-Diazacarbazole Analogs As Checkpoint Kinase 1 Inhibitors By Using 3D QSAR, Molecular Docking Study, and Molecular Dynamics Simulations

Xiaodong Gao, Liping Han and Yujie Ren

The structure of all the aligned molecular dataset used for the 3D QSAR studies were shown in Figure 1A. The 3D structures of the studies molecules were drawn and the 3D-QSAR studies were performed in SYBYL-X 2.0.

Table S1. Chemical structural formulas of all structures.

No.	2D Structure	3D Structure
1	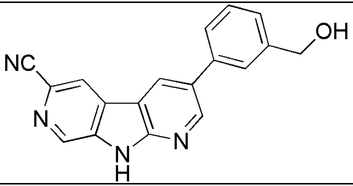	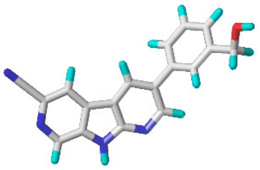
2	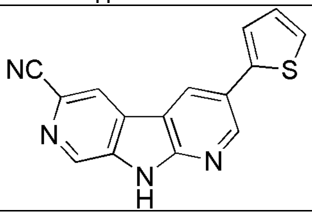	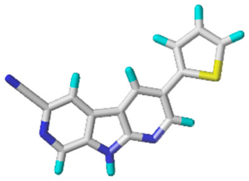
3	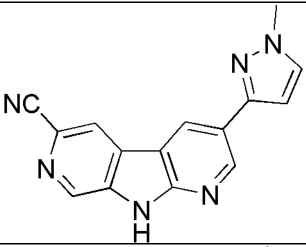	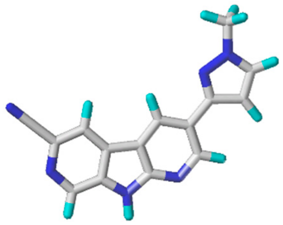
4	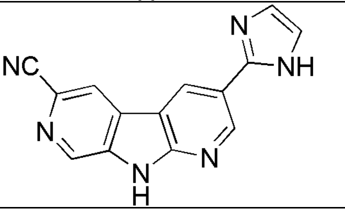	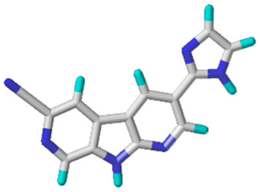
5	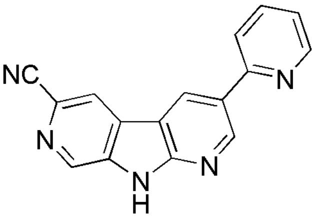	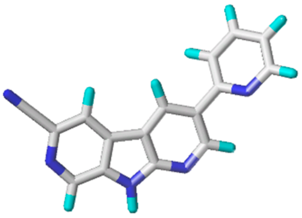
6	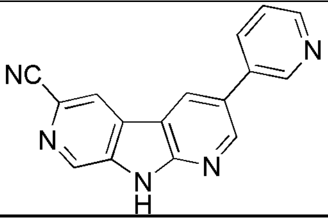	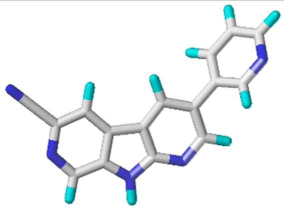

7	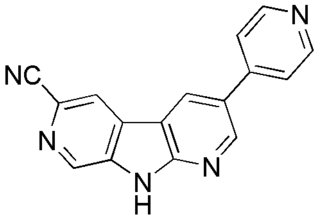	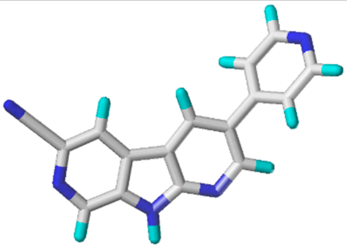
8	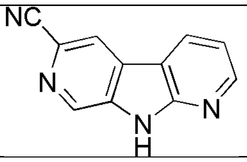	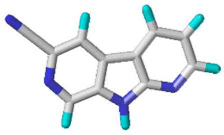
9	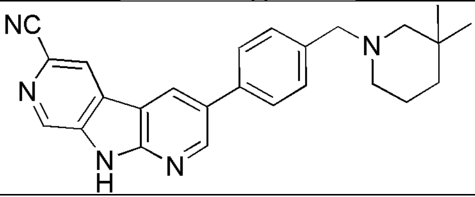	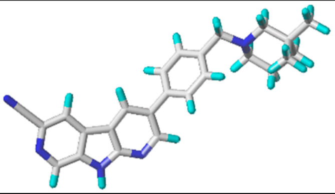
10	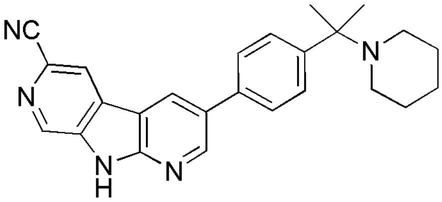	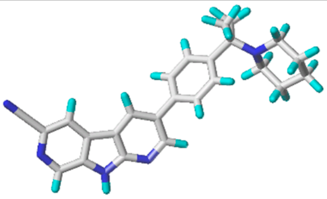
11	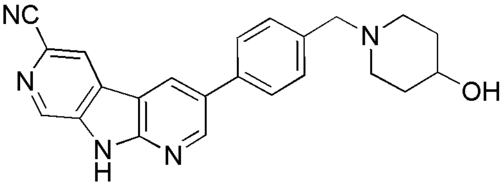	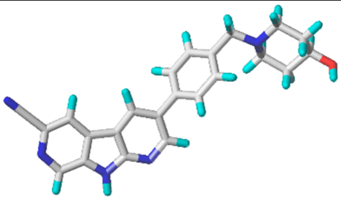
12	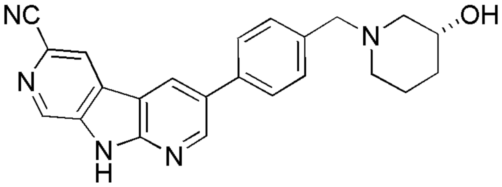	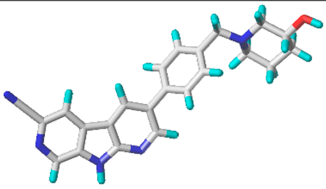
13	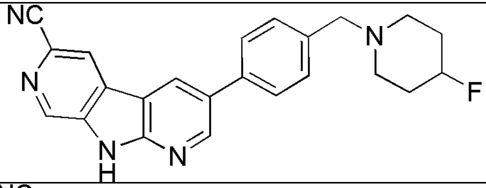	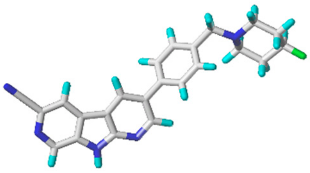
14	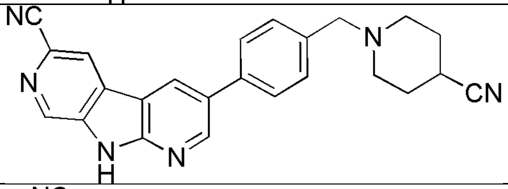	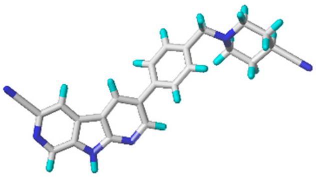
15	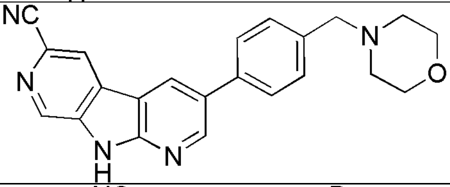	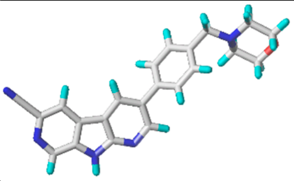
16	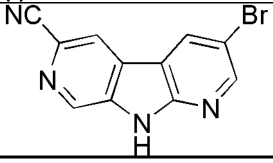	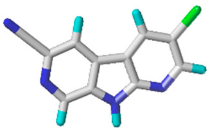

17	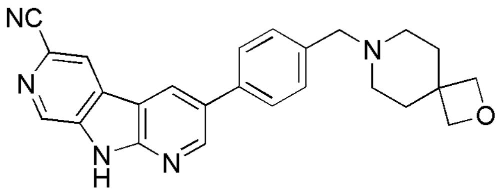	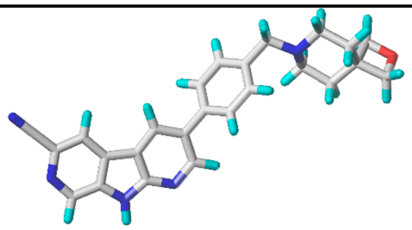
18	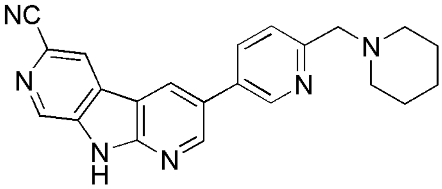	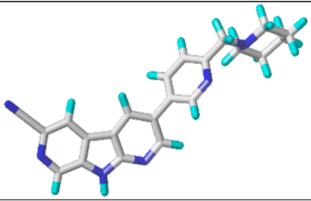
19	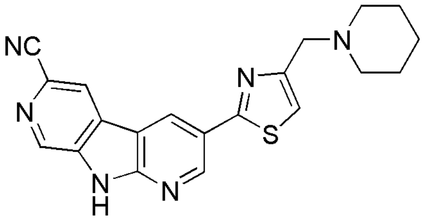	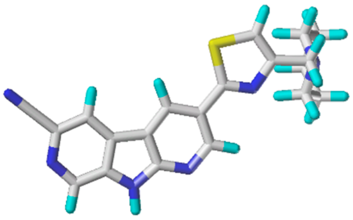
20	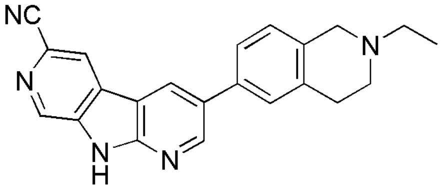	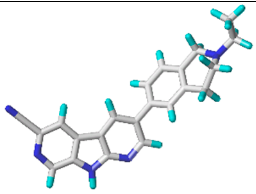
21	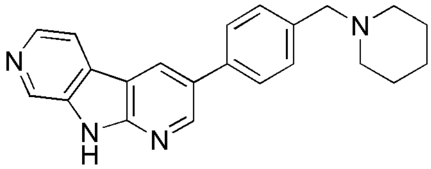	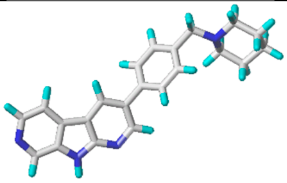
22	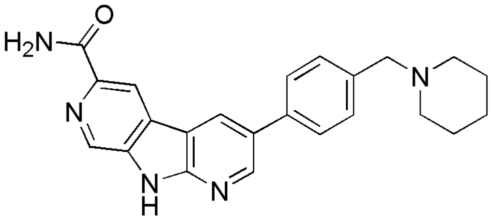	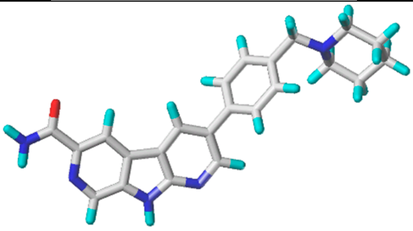
23	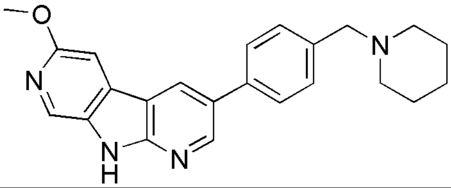	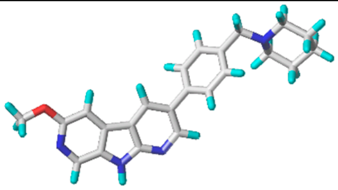
24	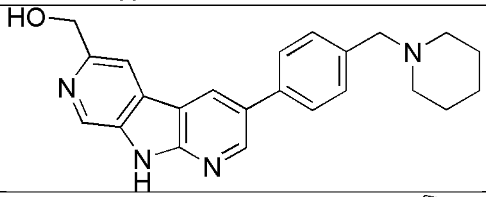	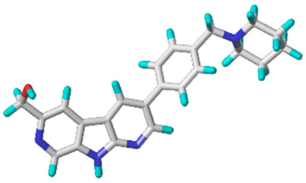
25	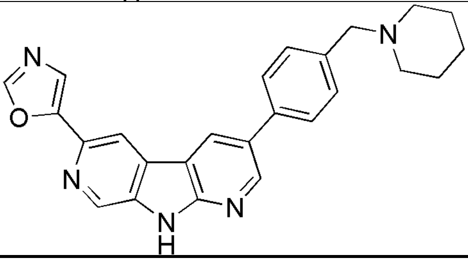	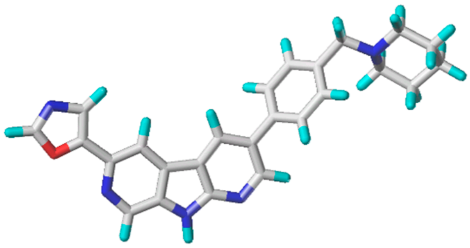

26	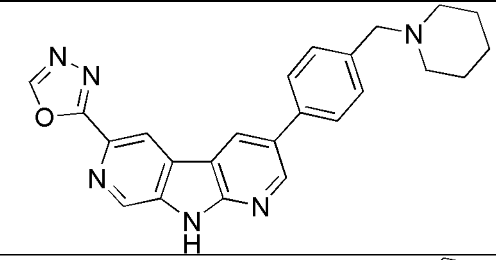	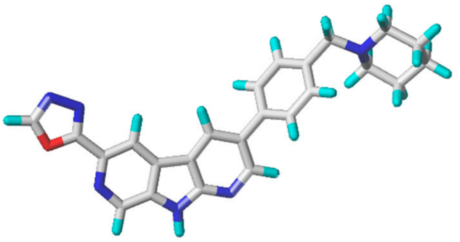
27	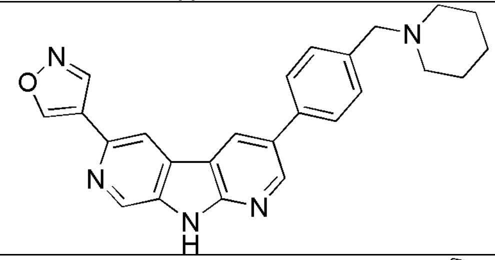	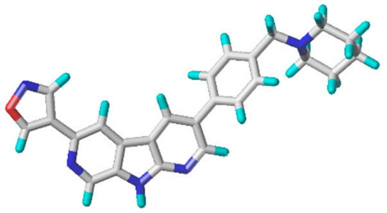
28	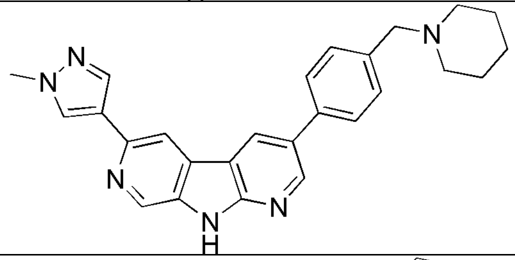	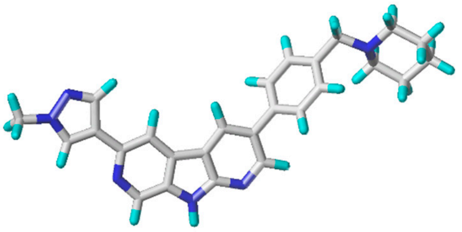
29	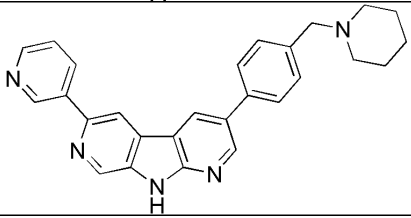	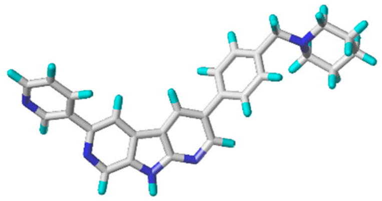
30	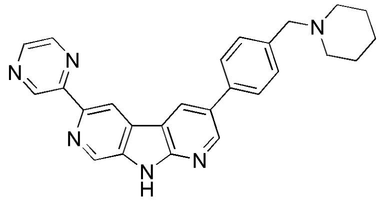	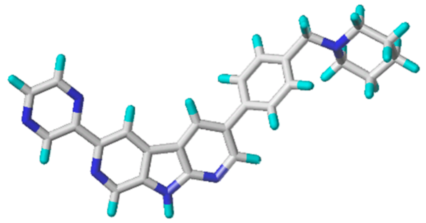
31	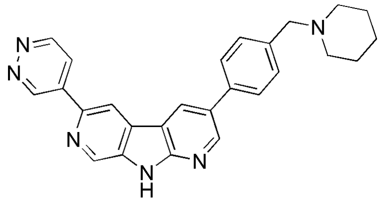	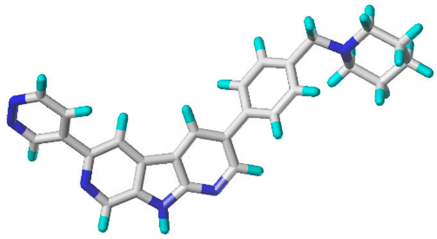
32	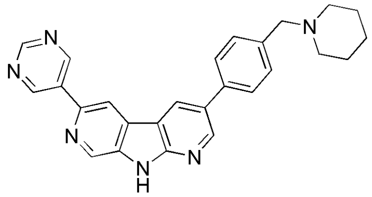	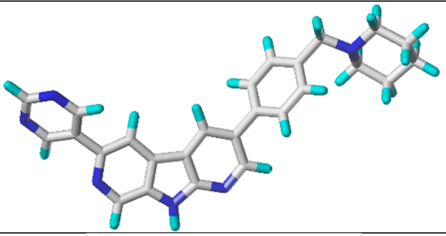
33	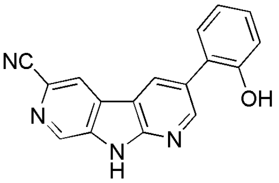	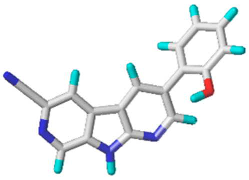

34	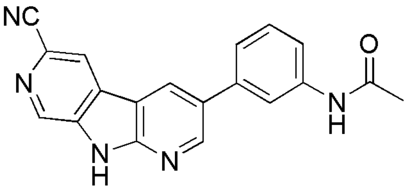	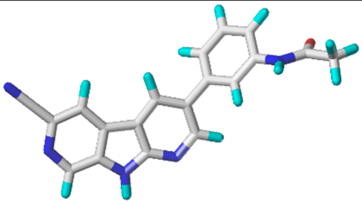
35	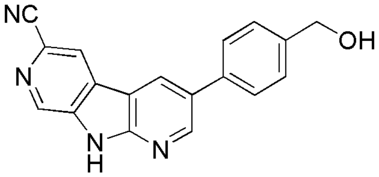	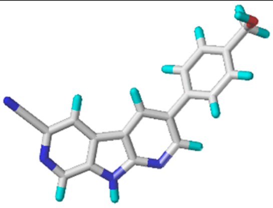
Test1	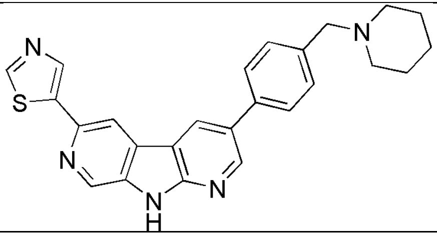	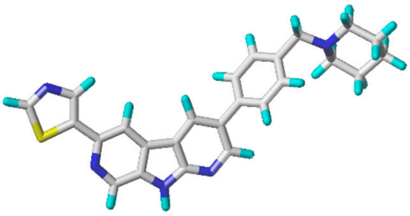
Test2	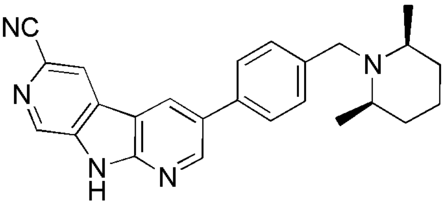	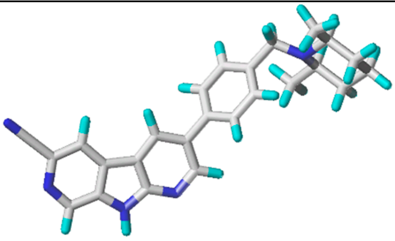
Test3	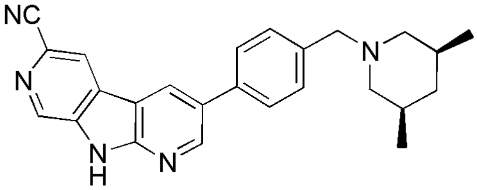	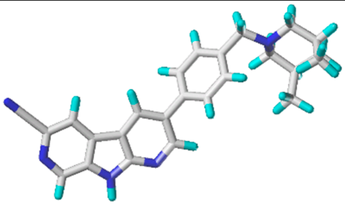
Test4	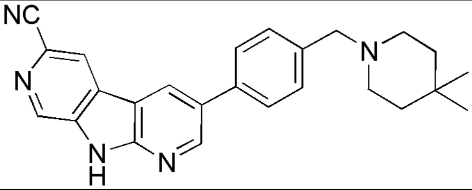	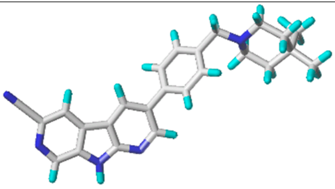
Test5	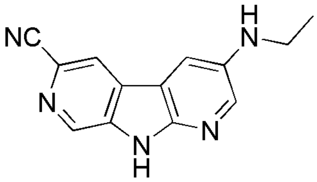	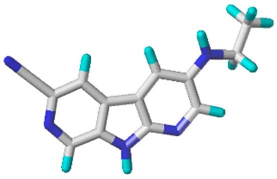
